# A Yoga Intervention for Posttraumatic Stress: A Preliminary Randomized Control Trial

**DOI:** 10.1155/2015/351746

**Published:** 2015-08-20

**Authors:** Farah Jindani, Nigel Turner, Sat Bir S. Khalsa

**Affiliations:** ^1^Centre for Addiction and Mental Health, 33 Russell Street, Toronto, ON, Canada M5S 2S1; ^2^Brigham and Women's Hospital, Harvard Medical School, 900 Commonwealth Avenue, Boston, MA 02215, USA

## Abstract

Yoga may be effective in the reduction of PTSD symptomology. The purpose of this study was to evaluate the impact of a Kundalini Yoga (KY) treatment on PTSD symptoms and overall wellbeing. To supplement the current field of inquiry, a pilot randomized control trial (RCT) was conducted comparing an 8-session KY intervention with a waitlist control group. 80 individuals with current PTSD symptoms participated. Both groups demonstrated changes in PTSD symptomology but yoga participants showed greater changes in measures of sleep, positive affect, perceived stress, anxiety, stress, and resilience. Between-groups effect sizes were small to moderate (0.09–0.25). KY may be an adjunctive or alternative intervention for PTSD. Findings indicate the need for further yoga research to better understand the mechanism of yoga in relation to mental and physical health, gender and ethnic comparisons, and short- and long-term yoga practice for psychiatric conditions.

## 1. Introduction

Posttraumatic stress disorder (PTSD) is a global health issue [1]. Epidemiologic studies indicate that approximately 8% of Americans have had or will have PTSD during their lifetime. Van Ameringen et al. [2] suggest that, at any given time, 2.4% of the population is experiencing PTSD symptoms. Women are twice as likely as men to develop PTSD [3]. Estimates suggest that trauma-related disorders cost over $45 billion U.S. dollars a year in medical and related costs [1, 3].

A vast research literature documents the physiological effects of PTSD [4–7]. Trauma is often followed by dysfunction in the stress response and emotion regulatory systems. Symptoms of PTSD may include, but are not limited to, rapid heartbeat, difficulty in breathing, muscle tightness, hyperarousal, inability to relax, chronic pain, mood issues, racing thoughts, and substance abuse and process addictions like problem gambling [8]. Individuals with PTSD often fail to demonstrate appropriate emotional responses because they are engaged in a pattern of reliving their prior trauma. Unable to move beyond their past trauma, these individuals are often disengaged from their present circumstances. Low self-esteem, a lack of coping skills, and an inability to relax due to high states of arousal are also common symptoms [7].

Within the medical community, there is a growing interest in studying the physiological and psychological effects of mind-body interventions, such as mindfulness and yoga, that encourage learning to focus attention on the present moment without judgment of thoughts, feelings, and emotions. Yoga focuses on breathing and physical exercises that combine movement, muscle relaxation, and meditation [9]. Mind-body practices may enhance the ability of a person with PTSD to tolerate unpleasant feelings and reduce stress [10–13]. Streeter et al. [13] suggest that yoga reduces stress-induced allostatic load in three stress reactive systems: the autonomic nervous system (ANS), the hypothalamic-pituitary adrenal (HPA) axis, and the GABAergic system. These findings are critical as they address the physiology associated with PTSD.

An essential component of trauma recovery is emotional regulation. Hölzel et al. [14] suggest that practices which encourage mindful awareness increase acceptance and tolerance of emotions thereby improving emotional regulation and normalizing perceptions of stress, critical for trauma recovery. Khalsa [15] concludes that yoga practice can increase resilience and enhance mind-body awareness, which may contribute to changing cognitions and behaviors. Mitchell et al. [16] conducted a 12-session Kripalu-based yoga intervention for PTSD with an assessment control group and van der Kolk et al. [17] demonstrated reduced PTSD symptomology in a sample of women participating in a hatha yoga treatment compared to a health education group. Both studies report positive findings but further research is required.

Kundalini Yoga as taught by Yogi Bhajan (KY) is a physical movement practice that includes meditation. Empirical research has reported that yoga aids in increasing awareness of one's physiological and psychological state [18, 19]. One KY meditation technique, Kirtan Kriya, has been found to increase cerebral blood flow and cognitive function [20]. Another KY practice has been found to significantly increase MRI signals in putamen, midbrain, pregenual anterior cingulate cortex, and hippocampal/parahippocampal formation [21].

KY has demonstrated that effectiveness greater than drug treatment in treating depression [22] is comparable to cognitive behavioral therapy in managing stress [9] and may be useful in the treatment of obsessive-compulsive disorder, insomnia, anxiety and impulse disorders like problem gambling [15, 23]. Empirical research has not examined KY as a treatment for posttraumatic stress. Randomized control trials examining the efficacy of yoga for PTSD are in their infancy. Rigorous trials are required. PTSD also affects overall health and lifestyle. To date, yoga and PTSD RCTs primarily focus on women and do not include additional measures of wellbeing.

The present study piloted an 8-week KY trauma program whereby participants were randomly assigned to either waitlist control or yoga group. We hypothesized that, through KY, physiological and psychological changes might occur, reducing PTSD symptoms. The overall goal of this study was to evaluate the impact of KY yoga intervention on symptoms of PTSD and resilience, positive and negative affect, mindfulness, insomnia, perceived stress and depression, anxiety, and stress.

## 2. Methods

### 2.1. Participants

Adults (9 males, 71 females) living within the community were recruited via (1) advertisements posted in the Greater Toronto area (GTA), (2) an online bulletin for patients at the Centre for Addiction and Mental Health (CAMH), and (3) advertisements posted at social services in the GTA.

80 participants were randomized to the yoga (*n* = 59) or control (*n* = 21) groups. 41 years (range 18–64 years) was median participant age. Study participants were exposed to a range of traumas (see Table 2).

Participants were only permitted study participation in any outside treatment if it did not have a contemplative component. These included CBT and exposure therapies. Approximately 57% of the waitlist control group sought alternative treatment while 39% of the yoga group was involved in other therapies. This difference was nonsignificant, *χ* ^2^ = 2.8, NS. (See Table 1 for participant descriptions.)

The PTSD cut-off score on the Posttraumatic Stress Disorder Checklist (PCL-17) is 57. Inclusion criteria were a score above 57 on the PCL-17 and 18+ years of age. Exclusion criteria included a regular contemplative practice, an inability to abstain from alcohol or substance 24 hours prior to class, or issues that would be a participant safety risk. No participants were denied study participation for reasons of safety or substance use.

Ten individuals did not begin the study due to scheduling conflicts, 8 participants were not able to complete the program due to medical and health reasons, 4 participants discontinued for personal reasons, and 8 participants had schedule changes, missed classes, or were on vacations eventually leading to study dropout. This resulted in a 30% dropout rate with 29 participants completing the yoga program and 21 in the waitlist control group.

### 2.2. Measures

Seven KY treatment group measures were acquired at baseline, midtreatment, and end-of-treatment to evaluate the impact of KY on dealing with issues such as PTSD, resilience, positive and negative affect, mindfulness, insomnia, perceived stress, depression, stress, and anxiety.

The Posttraumatic Stress Disorder Checklist (PCL-17) is a validated 17-item self-report scale [24]. Cronbach's alpha has ranged from 0.94 [24] to 0.97 [25], and the test-retest reliability was 0.96 at 2-3 days and 0.88 at 1 week [24].

The 25-item Resilience Scale (RS) measures the degree of individual resilience. Scores range from 25 to 175 with higher scores indicative of higher resilience. The RS demonstrates internal consistency (*r* = 0.91) and concurrent validity with established measures of adaptation such as morale (*r* = 0.28), life satisfaction (*r* = 0.30), and depression (*r* = −0.37) with Cronbach's alpha of 0.94 [26].

The Positive and Negative Affect Schedule (PANAS) is a 20-item psychometric scale that demonstrates relations between positive and negative personality traits [27]. For the Positive Affect Scale, the Cronbach alpha coefficient was 0.86 to 0.90 and for the Negative Affect Scale was 0.84 to 0.87. Over an 8-week period, the test-retest correlations were 0.47–0.68 for the PA and 0.39–0.71 for the NA [27]. The PANAS scale reports strong validity with measures of general distress and dysfunction, depression, and state anxiety [27].

The 5-Facet Mindfulness Questionnaire (FFMQ) is a 39-item self-report questionnaire assessing various aspects of mindfulness (e.g., observing, describing, being actively aware of present-moment experience, being nonjudgmental, and nonreactive focus). The five subscales have an internal consistency from 0.75 to 0.91 [28].

The Insomnia Severity Index (ISI) is used to detect cases of insomnia in population and clinical samples. Excellent internal consistency has been found for this measure (Cronbach *α* 0.91).

The Perceived Stress Scale (PSS) is the most widely used psychological instrument for the measurement of perceptions of stress with Cronbach's alpha value (0.82) [29].

The Depression, Anxiety, and Stress Scale (DASS 21) assesses distress along 3 dimensions: depression, anxiety (i.e., psychological arousal), and stress (cognitive or subjective anxiety symptoms). The internal consistency coefficient values (Cronbach's alpha) of each subscale range between 0.81 and 0.97 [30].

### 2.3. Procedure

**Figure 1:**
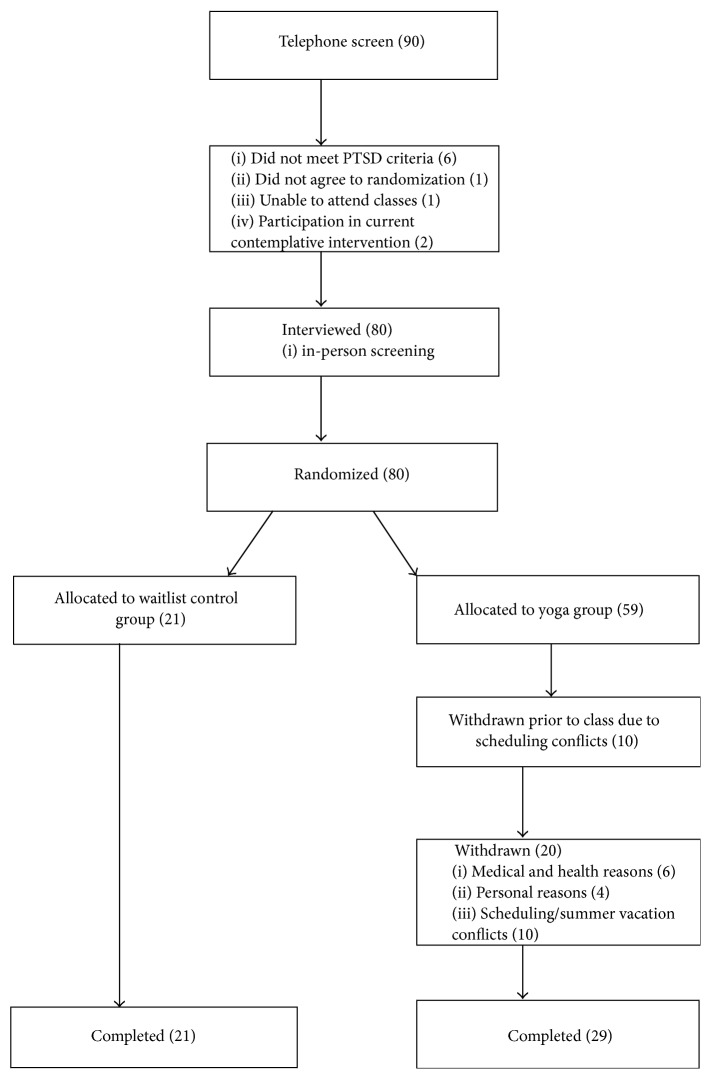
Flow of participants in study. PTSD = Posttraumatic Stress Disorder.

The University of Toronto office of research ethics approved study procedures. Study consent, recruitment, and facilitation occurred February–August 2012. Participants were screened by telephone (Figure 1) to assess entry criteria. The lead researcher (Farah Jindani) explained study purpose and logistics. Demographic information (i.e., age, symptomology, mental health diagnosis, prior treatment, medication, and prior contemplative experience) was collected.

Participants meeting entry criteria were assigned by a random number generator to either the experimental (yoga) group or the waitlist control. Waitlist participants participated in yoga at a later date. Both groups were 8 weeks in duration.

All participants underwent pretreatment baseline assessments of outcome variables through the University of Toronto online system. Midtreatment and final session questionnaires were completed in yoga class. Participants not able to attend class during weeks of data collection completed questionnaires online. Outcome measures for the waitlist group were completed online.

Yoga treatment groups participated in weekly 90-minute group training/practice sessions for 8 weeks and were encouraged to devote 15 minutes per day to a home practice. Three female certified KY teachers (International Kundalini Yoga Teachers Association) with over 10 years of teaching experience taught a total of 7 yoga groups ranging in size from 3 to 8 participants. Two of them each taught one class and the third teacher taught five classes. The yoga teachers had therapeutic mental health experience. A skilled KY teacher also attended each class as a support person.

### 2.4. Intervention

Kundalini Yoga (KY), a comprehensive yoga style incorporating the traditional elements of yoga practice including postures and physical exercises, breathing techniques, meditation, cultivation of mind-body awareness, and deep relaxation, was utilized in this study. The lead author in collaboration with a psychologist, a war veteran, and 2 KY yoga teachers developed the KY PTSD treatment protocol. All team members have a personal yoga practice. Curriculum exercises were chosen to address underlying causes of PTSD. Selected exercises and meditations are believed to help restore resilience and psychosocial integration through their effect on nervous and endocrine systems based on yogic philosophy.

The 8-week KY treatment protocol includes yoga practices specifically selected to help participants (a) develop the skills to relax and cope with trauma and related stress; (b) cultivate mindful awareness of the body, mind, breath, and environment; (c) improve cognitions, behavior, and emotions related to self-esteem and self-efficacy; (d) enhance flexibility, strength, and balance; and (e) reintegrate socially. Each 90-minute yoga class in the 8-week program consisted of the general class structure: active warm-up and loosening exercises, yoga postures and exercises, deep supine relaxation, yoga breathing techniques, meditation, and discussion of the physical, psychological, and philosophical principles of yoga.

Four KY yoga sets entitled Creating Internal Balance, Renew Your Nervous System and Build Stamina, Sahibi Kriya, and Adjust Your Flow were utilized in the curriculum. Each set was practiced for two consecutive weeks: week one at 1/2 time and the following week at full time (maximum of 45–50 minutes). Each weekly exercise and meditation in the protocol begin with small, manageable postures and meditations. As the individual's capacity increases, so does the length of time for postures and meditations. In this way, participants may gain skill and capacity for self-mastery.

In response to some of the unique challenges of trauma survivors, the protocol increases the time for deep relaxation over repeated practice. The protocol began with guided relaxation to support feelings of safety. Survivors of trauma may initially experience difficulty with quiet and stillness; therefore long periods of silent, unguided relaxation were not used initially.

A 15-minute daily home practice was integrated into the 8-week protocol. A 20-minute YouTube video [31] was created to make the support and instruction of a teacher available to participants as needed. The rationale for the home practice was that participants would learn tools to self-soothe in the program that they could utilize upon program completion.

The yoga program also integrated guidelines for trauma sensitive yoga [32] which emphasize inviting participants to try poses but never to stay in a posture that makes them uncomfortable. They were also encouraged to attend to thoughts and feelings without labeling them as good or bad, but learning to “be” with thoughts and feelings as they arise (curriculum available by request).

## 3. Results

Chi-square tests of independence were performed on all demographic categorical variables to determine the equivalency of the yoga and control groups. Table 1 demonstrates the raw scores and the calculated percentages of the waitlist control and yoga groups. Background characteristics were measured to determine if the samples were similar.

The only significant difference between the two groups was that males made up a slightly larger portion of the waitlist group before test. All of the males who began the study completed it.

The two groups were compared at baseline on all outcome variables using independent samples *t*-tests. At baseline, the only scale on which the differences between study groups reached statistical significance was the PCL-17 scores (*t*(78) = 2.39, *p* = 0.019). Mean scores (M) demonstrated that the intervention group had higher PTSD scores (PCL; M = 59.48, SD = 9.33) at baseline than the waitlist control group (M = 55.14, SD = 11.86).

The analyses were conducted using analysis of covariance (ANCOVA), which uses pretest scores to statistically control for differences between the groups. Repeated-measures ANOVAs with three time-points (baseline, mid-treatment, and end-of-treatment) were also performed on all outcome variables and findings were similar to the ANCOVA.

Change over time occurred across all groups and outcome measures. Means and standard deviations of questionnaire outcome data for the waitlist control and yoga treatment group across the three time intervals are presented in Table 3.

Therefore, the PCL-17 baseline scores were used as a covariate in further analyses (ANCOVA). ANCOVA results suggest that the assumption of equal slopes was checked and satisfied, as the interaction effect was not significant. Results of the ANCOVA found that the PCL scores were lower at follow-up for the yoga group compared to the waitlist control group *F*(1,48) = 15.64, *p* < 0.05, partial *n* ^2^ = 0.25. Similarly ISI scores for the waitlist control group were significantly lower than the yoga group at follow-up *F*(1,48) = 10.8, *p* < 0.05, partial *n* ^2^ = 0.18. PANAS (+) scores decreased for the waitlist control group and improved for the yoga group *F*(1,46) = 10.9, *p* < 0.05, partial *n* ^2^ = 0.19. PSS scores for the yoga group decreased by about half *F*(1,67) = 13.4, *p* < 0.05, partial *n* ^2^ = 0.17 in comparison to the waitlist control group, which decreased marginally. RS scores for the control group stayed about the same for the waitlist control group and increased significantly for the yoga group *F*(1,44) = 6.05, *p* < 0.05, partial *n* ^2^ = 0.12. DASS 21 scores decreased for both groups with greater change for the yoga group in the domains of anxiety, *F*(1,48) = 4.14, *p* < 0.05, partial *n* ^2^ = 0.08, and stress, *F*(1,48) = 4.69, *p* < 0.05, partial *n* ^2^ = 0.09. FFMQ scores improved marginally for both the waitlist control and yoga group but did not reach statistical significance.

## 4. Discussion

This study found that both the yoga group and the waitlist control groups changed over time in most outcome measures, although the yoga group had significantly greater improvements in scores of PTSD, insomnia, perceived stress, positive and negative affect, resilience, stress, and anxiety in comparison to the waitlist control. Participants in this program were not of a homogeneous trauma group and instead had experienced numerous adverse traumas. This suggests that the KY treatment intervention may serve as a beneficial adjunctive intervention for PTSD. Still, many questions remain regarding the benefits of yoga for psychiatric symptoms.

For instance, it is important to understand which elements of yoga practice influence reduction of symptoms and which styles of yoga are most beneficial for mental health issues. Further, while research has considered yoga to be an adjunctive treatment for PTSD, previous research has not considered whether yoga practices might serve as a helpful precursor to trauma-focused therapies. Yoga may reduce symptoms characteristic of PTSD particularly stress and arousal making one more prepared for insight based therapies that require emotional awareness. This finding is consistent with previous studies that have shown that yoga participants were better able to regulate emotions, focus on the present, and achieve restful states [13, 32].

The findings of this study suggest that when individuals feel calmer, they may experience greater awareness of their thoughts and emotions. Khalsa [15] postulates that awareness of the mind and body may be related to changes in thought patterns and behaviors. Other studies link self-awareness strategies to self-efficacy. Indeed, as the program progressed, participants demonstrated significant improvements in resilience.

This research adds to the limited RCT research base on the effects of yoga on PTSD using a modest sample size. Findings of this study also expand current research because significant differences were demonstrated between the yoga and control group in PTSD symptoms but also examined personality and lifestyle factors typically related to PTSD like insomnia, anxiety, perceived stress, and positive and negative affect. There are few published studies of yoga for PTSD and the majority of these have been symptom based. This is the first yoga RCT that examined symptoms as well as related lifestyle factors finding group differences. Finally, this is the first study examining PTSD using KY practices.

## 5. Limitations

While this study provides important data for future investigations and demonstrates the need for additional, larger RCTs, limitations should be considered. First the sample size was small and the study needs to be replicated with a larger number of participants before strong conclusion can be drawn. Second, the attrition in the yoga group likely affected the results. It is not known how much of the results were due to the differential dropout rate of the yoga versus waitlist group. Third, this intervention did not include a long-term follow-up that examined the seven outcome variables so it is not known if the group has any long-term impact on the participants' lives. Future research is needed to determine the impact of yoga on PTSD and related symptoms in the long-term. Fourth, although the participants were randomized to groups it was not possible to blind participants. Fifth, the control group was not an active control, but a waitlist. Because of the lack of blinding and the lack of an active control, it is not possible to separate the effects of Yoga from the effects of simply being part of a class and having weekly interaction with others. An active control such as a support group, for example, might have been able to test the potential unique effect of yoga as a therapy.

Yoga based interventions may be more appealing to some patients with PTSD than to others. While participants were diverse in terms of age, socioeconomic status, and physical fitness level, 85% of participants were Caucasian and the majority of the sample was female. While the sample was predominantly female, this is the only RCT for yoga that has included women and men. Diversity in terms of ethnic composition and balance in male/female ratios should be considered in future studies.

Simple questionnaires assessing the main areas of PTSD and overall wellbeing were included in this study to reduce participant burden. Further research covering a broad range of PTSD symptomology and factors related to mental health, wellbeing, and physiology are indicated. Future studies may consider using cognitive measures of attention or perception in addition to self-report psychometrics to identify PTSD and participant wellbeing.

## 6. Conclusion

This yoga study demonstrated significant changes in PTSD scores and other areas of wellbeing between the yoga and waitlist control groups. The findings of this KY PTSD study suggest that KY may be an adjunctive or alternative intervention for PTSD. Future work may examine gender, ethnic, and physiological variables. Further research is needed in order to understand the mechanisms behind apparent impact of yoga. Research is also required to determine the potential benefits to mental health from brief training in yoga as well as from long-term training in the practice of yoga.

## Figures and Tables

**Table 1:** Characteristics of study participants on baseline.

	Waitlist control	Yoga	*χ* ^2^
	*n* = 21	*n* = 59	
Gender			
Male	5 (24%)	4 (7%)	
Female	16 (76%)	55 (93%)	4.5^*∗*^
PTSD diagnosis	12 (57%)	38 (64%)	3.0
Therapy			
Current	12 (57%)	23 (39%)	
Past	4 (19%)	10 (17%)	
None	5 (24%)	26 (44%)	2.8
Yoga practice			
Past	1 (5%)	11 (19%)	
None	20 (95%)	48 (81%)	2.3
Meditative practice			
Past	0 (0%)	9 (15%)	
None	21 (100%)	50 (85%)	3.6
Medication			
Prescribed	9 (43%)	29 (49%)	
None	12 (57%)	30 (51%)	.25

^*∗*^ *p* < .05.

**Table 2:** Self-reported trauma of participants at study onset.

Type of trauma (*N* = 80)	*n*
Sexual abuse (including childhood sexual abuse)	13
Physical trauma (e.g., illness, motor vehicle accidents)	9
Emotional abuse	18
Domestic violence	7
Systemic discrimination (e.g., racism, heterosexism)	3
Compassion fatigue (e.g., vicarious trauma, secondary trauma)	2
Adverse life circumstances (e.g., employment, relationships)	12
Complex multiple traumas (e.g., family, refugee, chronic illness)	16

**Table 3:** Means and standard deviations for study outcomes by treatment group before treatment, at midtreatment, and after treatment.

	Waitlist control group						Yoga group					
Measure	Before treatment		Midtreatment		After treatment		Before treatment		Midtreatment		After treatment	
	*n* = 21		*n* = 21		*n* = 21		*n* = 29		*n* = 29		*n* = 29	
	M	SD	M	SD	M	SD	M	SD	M	SD	M	SD
PCL-17	55.1	11.9	52.5	11.6	55.4	13.5	59.5	9.3	48.5	14.3	41.8	12.0
ISI	16.1	7.9	16.5	5.6	16.4	5.8	14.4	8.1	11.4	8.0	10.6	6.7
PANAS (+ve)	26.9	7.9	24.5	7.0	23.8	6.1	26.3	7.5	28.6	6.8	30.5	7.9
PANAS (−ve)	24.7	9.3	25.7	9.2	21.9	7.7	24.2	9.4	22.4	9.0	19.0	7.7
PSS	24.8	7.2	21.8	6.6	21.6	4.8	24.9	7.6	15.4	12.0	12.4	11.4
DASS 21 (depression)	10.3	5.8	8.5	5.8	7.2	5.1	8.1	4.7	7.6	4.8	6.0	4.3
DASS 21 (anxiety)	9.6	5.4	9.3	5.5	7.8	5.5	9.4	5.2	7.1	5.0	5.7	4.3
DASS 21 (stress)	12.9	5.7	12.4	5.3	11.0	4.4	13.2	4.8	10.5	5.3	8.8	4.5
FFMQ (observing)	25.4	5.5	24.6	7.0	24.6	6.5	26.5	6.1	28.9	9.7	271	6.0
FFMQ (describing)	24.7	3.3	23.6	4.7	24.0	4.5	24.7	2.8	25.2	3.8	25.1	2.6
FFMQ (acting with awareness)	28.4	8.0	28.5	7.9	26.6	7.6	26.0	4.5	23.8	5.4	22.3	6.5
FFMQ (being nonjudgemental)	28.7	7.9	26.3	8.5	25.6	8.7	25.6	7.1	21.1	8.6	21.7	8.5
FFMQ (nonreactive focus)	18.9	5.9	20.0	5.7	20.3	3.3	18.2	4.9	20.1	5.0	21.1	4.9
RS	110.7	25.8	114.3	20.3	111.1	23.9	112.4	24.1	122.5	23.3	124.7	23.2

*Note*. PCL = PTSD Checklist; ISI = Insomnia Severity Index; PANAS = Positive and Negative Affect Schedule; PSS = Perceived Stress Scale; DASS 21 = Depression, Anxiety, and Stress Scale; FFMQ = 5-Facet Mindfulness Questionnaire; RS = Resilience Scale.
